# Interreg and WHO-CC : a Fruitful Collaboration in the Service of E-Mental Health

**DOI:** 10.1192/j.eurpsy.2022.105

**Published:** 2022-09-01

**Authors:** D. Sebbane

**Affiliations:** WHO Collaborative Center, Epsm Lille, Lille, France

**Keywords:** mental health, innovative technology, digital psychiatry, Anxiety

## Abstract

Within the IT4Anxiety project, the World Health Organisation Collaborating Centre (WHOCC) for research and training in mental health of Lille, is the leader of the work package “identification of needs among the different actors in the mental health field”. The complexity of our object of study - the use of new technologies for people’s mental health - requires undertaking a multi-disciplinary approach, considering both the clinical and the socio-anthropological aspects. A mixed-methods research conducted with pepole people experiencing anxiety, carers and professionnals, aims to describe the use, acceptability and needs of the different categories of actors concerned by the use of new technologies. The quantitative part is a cross-sectional, descriptive, observational study. A anonymous self-administered questionnaire is distributed online and in hard copy in the 5 partner countries (Belgium, France, Germany, the Netherlands and the UK) to the three study populations. It consists of access to digital tools or technological solutions, experience related to these and the contribution and perceived effect of users to e-mental health related projects. The qualitative approach includes a detailed understanding of the potential therapeutic and socio-anthropological changes that these tools can bring. It explores : description of used technologies, the representations and emerging needs in relation to the management of anxiety and the improvement of mental health ; the clinical impact perceived by all stakeholders and the implementation of new ways of living with the tools. This international research will enable a comparative study to be carried out on the dynamics of appropriation of these e-(mental) health devices in different countries of the North-Western European region.

**Disclosure:**

No significant relationships.

